# Characterization of Dendritic Cells Subpopulations in Skin and Afferent Lymph in the Swine Model

**DOI:** 10.1371/journal.pone.0016320

**Published:** 2011-01-27

**Authors:** Florian Marquet, Michel Bonneau, Florentina Pascale, Celine Urien, Chantal Kang, Isabelle Schwartz-Cornil, Nicolas Bertho

**Affiliations:** 1 Virologie et Immunologie Moléculaires UR892, Institut National de la Recherche Agronomique, Domaine de Vilvert, Jouy-en-Josas, France; 2 Centre de Recherche en Imagerie Interventionnelle, Institut National de la Recherche Agronomique, Domaine de Vilvert, Jouy-en-Josas, France; Institut Pasteur, France

## Abstract

Transcutaneous delivery of vaccines to specific skin dendritic cells (DC) subsets is foreseen as a promising strategy to induce strong and specific types of immune responses such as tolerance, cytotoxicity or humoral immunity. Because of striking histological similarities between human and pig skin, pig is recognized as the most suitable model to study the cutaneous delivery of medicine. Therefore improving the knowledge on swine skin DC subsets would be highly valuable to the skin vaccine field. In this study, we showed that pig skin DC comprise the classical epidermal langerhans cells (LC) and dermal DC (DDC) that could be divided in 3 subsets according to their phenotypes: (1) the CD163^neg^/CD172a^neg^, (2) the CD163^high^CD172a^pos^ and (3) the CD163^low^CD172a^pos^ DDC. These subtypes have the capacity to migrate from skin to lymph node since we detected them in pseudo-afferent lymph. Extensive phenotyping with a set of markers suggested that the CD163^high^ DDC resemble the antibody response-inducing human skin DC/macrophages whereas the CD163^neg^CD172^low^ DDC share properties with the CD8^+^ T cell response-inducing murine skin CD103^pos^ DC. This work, by showing similarities between human, mouse and swine skin DC, establishes pig as a model of choice for the development of transcutaneous immunisation strategies targeting DC.

## Introduction

Vaccines targeting skin, through intradermal or epicutaneous delivery, present several advantages compared to the vaccine given intramuscularly, such as: dose sparing [1] and better activation of cytotoxic and mucosal responses [2], [3]. Current knowledge supports that optimal induction of immune responses depends on the dendritic cell (DC) subtypes that are targeted by vaccines. Thus the identification of the DC subtypes in the skin and the analysis of their specialized function in immunity are key steps in the development of cutaneous delivered vaccines. Most information on skin DC has been obtained in the mouse model (for review see [4]). Mouse epidermis contains a unique DC subset, the Langherans cells (LC), that can be identified in the mouse by their high expression of Langerin (CD207). Some migrating LC, en route for the lymph nodes (LN), are also found in the dermis [5]. The role of LC is still unclear in the mouse, but converging results suggest that they behave as immunoregulatory cells [6], [7], [8]. Mouse dermal DC (DDC) comprise four distinct subsets, the discrete CD207^pos^CD172a^neg^CD103^pos^, the CD207^pos^CD172a^pos^CD103^neg^, CD207^neg^CD172a^pos^CD11b^high^ and CD207^neg^CD172a^pos^CD11b^neg^ DDC subsets [5]. The CD207^pos^CD172a^neg^CD103^pos^ DDC have received much attention recently as they play a key role in cross-presentation for tolerance induction and in mounting a CD8^+^ T cell immune response [5], [8],[9]. Although playing a major role in specific immune responses, they represent less than 5% of the DDC [5], [10], but 13% of the skin draining LN DC [5].

Much less data are available for the human skin DC. Human CD207^high^ LC have been identified in epidermis, but contrary to mouse, they were described as the most efficient skin DC subset to expand antigen specific CD8^+^ T cells by antigen cross-presentation [11]. Besides, two DC subsets (CD14^pos^ and CD1a^pos^ DDC) were distinguished in the human dermis [11], [12], but they do not share cell surface phenotype with the mouse DDC. For instance, the CD207 marker was not found expressed by human DDC. The minor CD14^pos^ DDC subset expresses several macrophagic markers such as CD163, DC-Sign/CD209 and the mannose-receptor/CD206, and was found to prime CD4^pos^ T cells into cells that induce isotype switching in B cells. The largely represented CD1a^pos^ DDC subset was revealed to activate CD8^+^ T cells better than CD14^pos^ DC but less efficiently than LC.

Results on human skin DC functions were generated *in vitro*, whereas the mouse DC functional data were obtained *in vivo*. These different experimental approaches may explain the discordant functional results obtained from the two species. Alternatively, intrinsic differences in the mouse and human skin structures such as the hairiness and the stratum corneum thickness might be associated to evolutionary divergences in DC subpopulation roles. Evaluation of novel vaccine strategies *via* the skin would thus greatly benefit from a more relevant animal model that would permit *in vivo* investigation. Pig skin shares strong histological similarities with human skin, such as low hairiness, thick strateum corneum with similar lipid composition [13], and dermis structure [14]. CD207^high^ LC have been identified in pig epidermis [15], [16], [17]. In addition, pig DC that had migrated from skin explants expressed CD1, CD172a, MHC-II and CD80/CD86 [15]. Finally, pig is a large mammal which permitted us to adapt for the first time pseudo afferent catheterism to swine skin lymph collection [18].

In this report, we thoroughly described and analyzed 4 swine DC subsets in epidermis, dermis and lymph, and we suggested possible correspondences with mouse and human skin DC. This provides a first analytical and dynamic picture on the swine skin DC establishing pig as a relevant model to study skin DC subsets role in immune responses and to develop novel vaccine strategies.

## Results

### Selection of the markers used for the discrimination of DC subtypes

**Figure 1:**
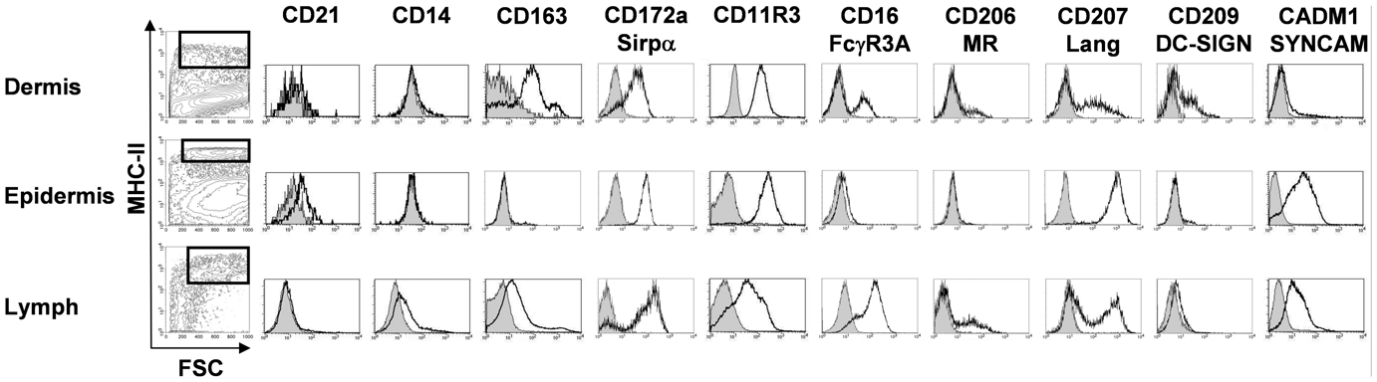
Phenotyping of swine DC in skin and afferent lymph. FACS analysis of DC from dermis, epidermis and pseudo-afferent iliac lymph. Skin biopsies were first split in epidermis and dermis by dispase digestion of the basal layer, before overnight collagenase digestion of the dermis. Lymph DC were enriched by density gradient before staining. Dot-plots represent the gating strategy (FSC^high^/MHC-II^high^ cells) used to electronically isolate DC from the different tissues. Histograms depict isotype control (plain histograms) versus indicated marker expression (bold, open histograms) and are representative of at least 3 independent experiments on 3 different animals.

For FACS gating of DC from skin and lymph, we used the most widely recognized phenotypic definition of DC, as being large, MHC-II^high^ cells. We first checked, using MHC-II/CD14 staining, that CD14^high^ dermal macrophages were not present in the MHC-II^high^ gate; similarly MHC-II/CD21 staining assured us that CD21^pos^ dermal B lymphocytes were absent from the DC gate (Figure 1). We then tested a panel of antibodies available in pig allowing the detection of several proteins previously described on mouse and human DC (Figure 1). These markers were CD163 (the scavenger receptor of haemoglobin-haptoglobin-complexes), CD172a/Sirpα, the ligand of CD47, CD11R3, a possible pig equivalent to CD11b [19], CADM1/SYNCAM, described as differentially expressed in cross-presenting DC in mouse [20] and sheep [21], and having a potential role in DC/CD8 T cells interaction [22], [23], CD16/FcγR3A, CD206/Mannose Receptor, CD207/Langerin and CD209/DC-Sign. The expression of each antigen was first tested on DC extracted from dispase-split epidermal and dermal sheets with or without collagenase digestion. Indeed, the use of collagenase allowed the collection of up to 30 times more DDC. We observed no variation, nor in percentage of expressing cells, neither in expression intensities of all the markers tested at the exception of CadM1, whose expression was totally abrogated by the collagenase treatment (data not shown). Thus CadM1 expression was analysed on DC extracted from split epidermis and dermis in absence of collagenase.

DDC presented bi-modal expression patterns of CD172a, CD16, CD206, CD207/Lang and CD209/DC-Sign, an even more complex expression of CD163, which discriminate 3 different DDC populations (negative, low and high CD163 expressing DC), a homogenous expression of CD11R3 and almost no CadM1 detection. Epidermal DC were homogenous in the expression of all the markers tested, at the exception of CD209, which discriminated about 5% of positive cells from the main CD209^neg^ population. We observed no expression of CD163 and CD206; low to null expression of CD16, depending of the animal; and a strikingly high expression of CD207.

In order to analyze the skin DC that migrate in lymph, lymph duct catheterism was conducted. FSC/SSC^high^ and MHC-II^high^ cells, represented around 1% of the total lymph cells. We next characterized lymph DC, they presented similar complex expression patterns as DDC for all the tested antigens, at the exception of CD209 which was not expressed on lymph DC.

### Definition and phenotype of four DC subtypes

**Figure 2:**
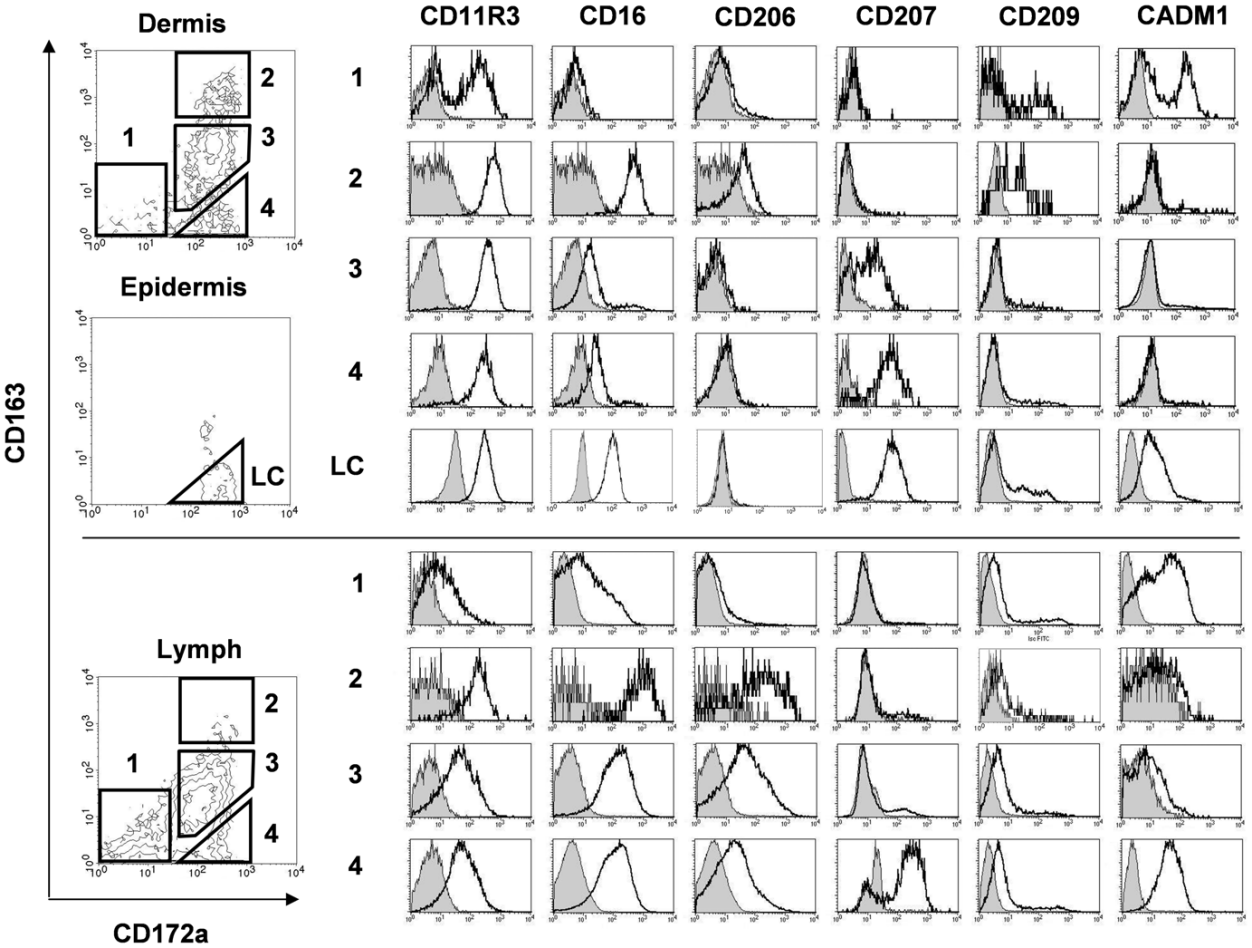
Characterization of the 4 DC subsets present in skin and lymph. FACS analysis of DC from dermis, epidermis and pseudo-afferent iliac lymph. DC were gated using the same criterions as in Figure 1 (FSC^high^/MHC-II^high^). The expressions of the markers identified in Figure 1 were differentially reexamined on lymph and dermal DC subpopulations characterized as CD163^neg^/CD172a^neg^ (1), CD163^high^/CD172a^pos^ (2), CD163^low^/CD172a^pos^ (3), and CD163^neg^/CD172a^pos^ (4). In epidermis, Langerhans cells (LC) were retrieved in the same gate than population (4) from dermis and lymph. Histograms depict isotype control (plain histograms) versus indicated marker expression (bold, open histograms) and are representative of at least 3 independent experiments on 3 different animals.

The complex marker expression patterns found on lymph and dermal DC suggested the existence of DC subpopulations. In human skin, CD163 has been described on a macrophage-like DDC subpopulation [11], [12], [24] whereas CD172a^neg^ DC have been described in several species as endowed with cross-presentation capacities [5], [8], [9], [21]. We thus chose to phenotypically characterized DC subpopulations distinguished by the expression of CD163 and CD172a (Figure 2).

Using CD163 and CD172a, 4 subpopulations could be segregated in the dermis: the quantitatively minor population (1) CD163^neg^CD172a^neg^ (9+/- 2%), the population (2) CD163^high^CD172a^pos^ (25+/- 3%), the population (3) CD163^low^CD172a^pos^, constituting the majority of the DDC (47+/- 7%) and the population (4) CD163^neg^CD172a^pos^ DDC (11+/- 5%). Population (1) was negative for the majority of the markers tested, with the exception of a bimodal expression of CD11R3 (CD11R3^neg^ and CD11R3^low^) and CadM1 (CadM1^neg^ and CadM1^low^) and a minor CD209^pos^ subpopulation. The expression of CadM1 in this subpopulation (1) is in agreement with the described CadM1^pos^/CD172a^neg^ phenotype of cross-priming DC in mouse spleen [20] and sheep afferent lymph [21]. The CD163^high^ DDC population (2) presented a strikingly high expression of CD16 and was positive for CD206 and CD209, in agreement with a ‘macrophage like’ phenotype [11], [12], [24]. The DDC population (3) was CD16^low^ and presented a heterogeneous expression of CD207/Langerin. Finally, in the dermis the last population (4) highly expressed CD207. The epidermis presented one unique DC population, namely Langerhans cells (LC), constituting the vast majority of the epidermal DC (95+/- 3%). They expressed the same levels of CD163 and CD172a than the dermal DC population (4). Moreover, with the exception of CD16 which is lower, and of CadM1 which is not expressed in the DDC population (4), LC and DDC population (4) expressed the same levels of the other markers tested, pleading for a common epidermal origin of these two DC populations.

In the lymph, the same 4 DC subpopulations were observed, however their distribution was quite different from skin (Table 1) since the minor dermal DC population (1) CD163^neg^/CD172a^neg^ represented 26+/- 3% of the lymph DC, whereas the subtype (2) CD163^high^/CD172a^pos^, well represented in the dermis, represented only 2+/- 2% of the lymph DC. The lymphatic distribution of these two subpopulations is significantly different from the distribution of their skin counterparts (p<0.001). The lymphatic distributions of populations (3) and (4) were not different from skin (respectively 52+/- 8% and 13+/- 8%).

**Table 1:** Skin and lymph DC sub populations.

			% (+/- SD)	CD11R3	CD16/FcγR3	CD206/MR	CD207/Lang	CD209/DCSign	CadM1/SYNCAM
**1**	**CD163^neg^/CD172a^neg^**	**Dermis**	9% (+/- 2%) *	++ and -	-	-	-	-	++ and -
		**Lymph**	26% (+/- 3%) *	+	+/-	-	-	+/-	++
**2**	**CD163^high^/CD172a^pos^**	**Dermis**	25% (+/- 3%) *	+++	**+++**	++	-	++	+/-
		**Lymph**	2% (+/- 2%) *	+++	**+++**	+	-	+/-	-
**3**	**CD163^low^/CD172a^pos^**	**Dermis**	**47% (+/- 7%)**	+++	+	-	+ and -	-	-
		**Lymph**	**52% (+/- 8%)**	++	++	+	+ and -	+/-	-
**4/LC**	**CD163^neg^/CD172a^pos^**	**Epidermis**	**95% (+/- 3%)**	+++	++	-	**+++**	+ and -	++
		**Dermis**	**11% (+/- 5%)**	+++	+	-	**+++**	-	-
		**Lymph**	**13% (+/- 8%)**	++	++	+	**+++**	+/-	++

Four DC subpopulations have been characterized in lymph and skin. Percents +/- standard deviation (SD) represent the mean of at least 4 experiments on different animals.

*Represent significant differences (student T test p<0.001) in the percentage of DC subpopulations between dermis and lymph. Expression intensity for each antigen is depicted from (++++): Highly expressed, to (+/-): Very low expression, to (-): No expression at all. (+ and -) means that there is a bimodal expression of the antigen. The markers allowing the best discrimination between DC subtypes are highlighted in bold.

Lymph DC subpopulations expressed mostly the same markers than their skin counterparts. The most striking similarities were: i) the CadM1 expression of the CD172a^neg^ DDC population (1), ii) the CD16^high^ profile of the CD163^high^ DDC population (2), iii) the CD207^high^ expression of the DDC (4)/LC populations. The main differences between lymph and skin DC were the higher expression of CD16 on CD163^low^ lymph DC (populations (3) and (4)) compared to their skin counterparts, a higher general expression of CD206 and a more homogenous weak to null expression of CD209 in all the lymph DC subpopulations (Figure 2).

**Figure 3:**
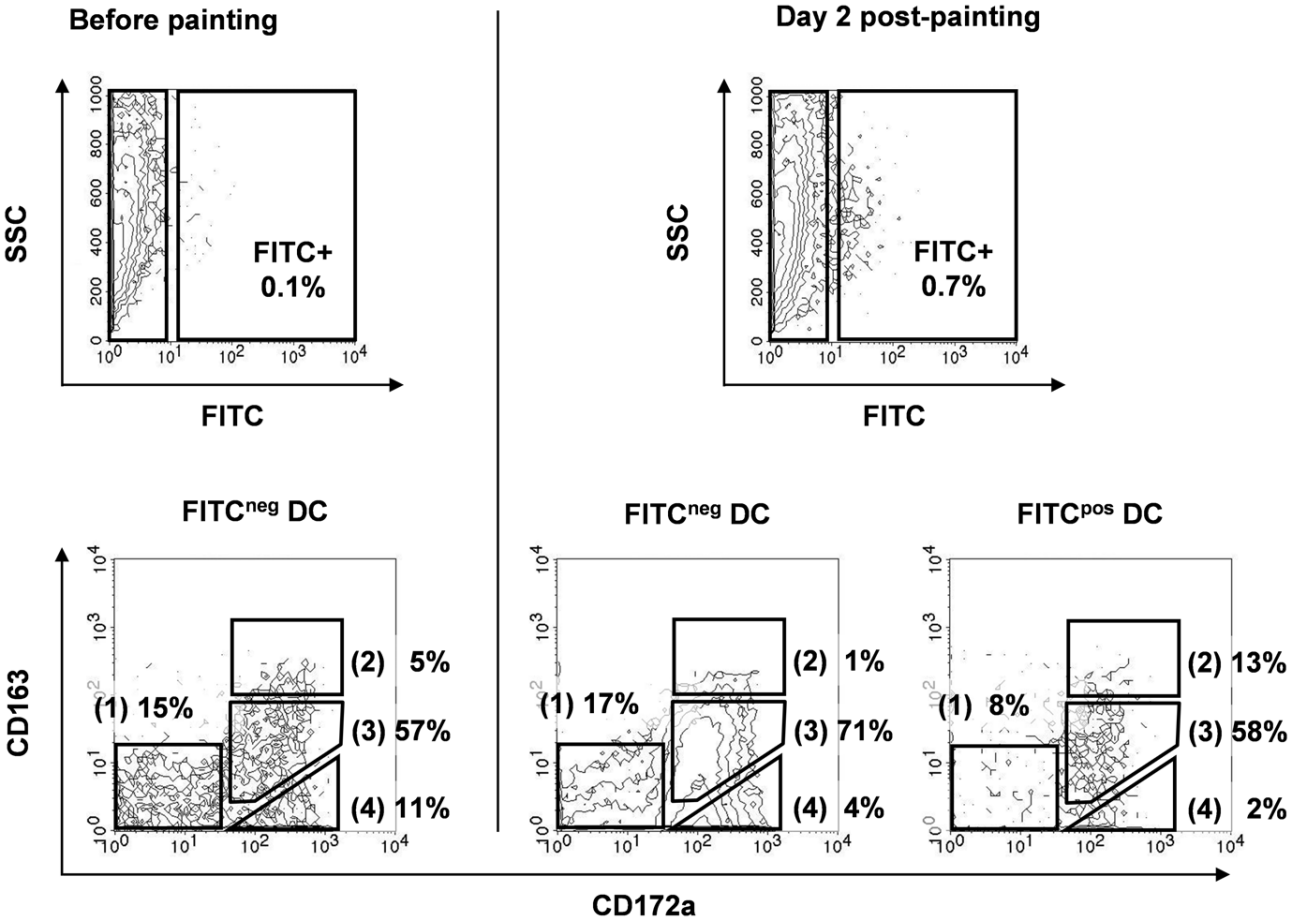
Skin FITC painting attests that all the lymph DC subtypes migrate from skin. Skin FITC painting allows the detection of DC migrating from skin to lymph. FITC at 1 mg/ml in acetone/dibutylphtalate (V/V) was applied on the leg of the animal, lymph was collected before painting and 2 days after painting, and lymph cells were then stained for MHC-II, CD172a and CD163. DC were gated using the same criterions as in Figure 1 (FSC^high^/MHC-II^high^). FITC^neg^ and FITC^pos^ DC subpopulations are depicted. Data are representative of two independent experiments.

In order to demonstrate that lymph DC subpopulations came from skin territories, we proceeded to skin FITC painting and we observed, after two days, lymph DC having incorporated FITC. The same lymph DC subpopulations (1), (2), (3) and (4) were observed in FITC^pos^ DC than in FITC^neg^ DC, although not exactly in the same proportions (Figure 3).

Thus, as emphasized in Table 1, we described here 4 different skin DC subtypes and followed their *in vivo* migration from skin to LN, through the afferent lymphatic.

### In situ characterization of skin DC

**Figure 4:**
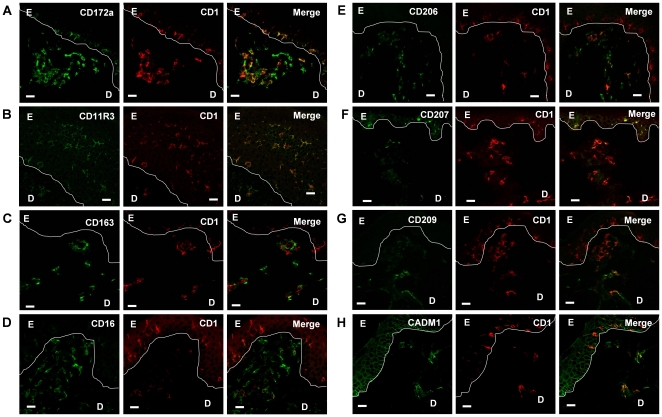
*In situ* skin DC phenotyping. Sections of normal swine skin were acetone/methanol fixed and stained for the indicated specificity (green) and CD1 (red). A. CD172a, B. CD11R3, C. CD163, D. CD16, E. CD206, F. CD207, G. CD209, H. CADM1. Antibodies used are described in Materials and Methods. Images are representative of at least 5 pictures from different regions of biopsies from at least 3 different animals, independently stained. Objective used 40x oil immersion. E: Epidermis, D: Dermis, Scale bars  = 10 µm.

In confocal experiments, anti-CD1 antibodies appeared to more distinctly stain DC than anti-MHC-II antibodies. Moreover we and others [15] have checked that MHC-II and CD1 molecules were co-expressed on the vast majority of skin cells. We thus used CD1 instead of MHC-II to detect skin DC *in situ*. CD1 staining allowed the unambiguous distinction between skin DC and macrophages, since preliminary experiments showed that skin macrophages were CD1^neg^/CD14^high^ whereas skin DC were CD1^pos^/CD14^neg/low^ (data not shown). Using CD1 or MHC-II as DC markers, we were not able to distinguish CD172a^neg^ from CD172^pos^ DC (Figure 4A), however CadM1 expression (Figure 4H), a signature of CD172a^neg^ DC, was observed in the dermis. CD11R3 appears highly expressed on DDC and on LC (Figure 4B). As expected from flow cytometry analysis CD163, CD206 and CD209 were observed on a fraction of DDC, but not on LC (Figures 4C, E, G). CD16 was expressed on a DDC subpopulation, but also, at a weaker level, on LC (Figure 4D). As expected, CD207 was expressed on LC, but also on some DDC (Figure 4F). Finally CADM1 expression appeared in the epidermis on cells along the basal layer, corresponding to undifferentiated keratinocytes, but also on LC and on some DDC (Figure 4H). In addition, different staining combinations confirmed that CD163^pos^ DDC expressed CD16, CD206 and CD209, but neither CD207 nor CadM1 (data not shown). Thus we were able to detect *in situ* the 4 skin DC subpopulations defined by Facs analysis. Except for the LC, present in the epidermis, no preferential localization of the 3 DDC subpopulations in upper or lower dermis was observed.

### CD172a^neg^/CD163^neg^ DDC and lymph DC transport apoptotic bodies

**Figure 5:**
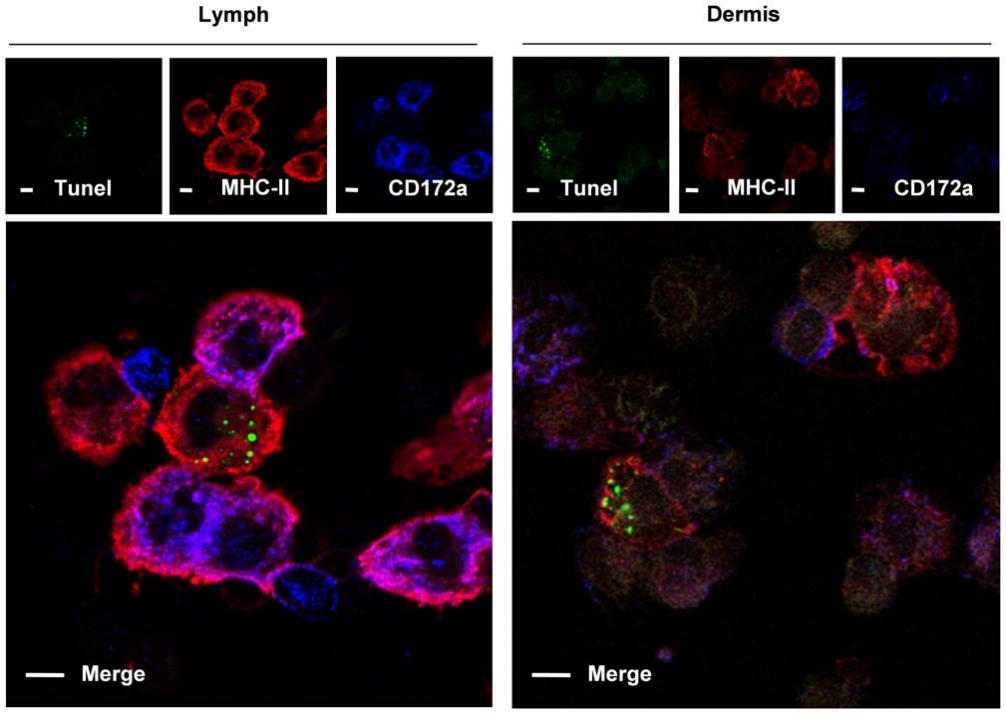
CD172a^neg^ DC from lymph and dermis contain apoptotic bodies. Lymph DC were partially enriched by low density gradient. Skin biopsies were first split in epidermis and dermis by dispase digestion, before overnight collagenase digestion of the dermis. Dermal DC were then enriched by Ficoll gradient. DC were further purified using MHC-II and magnetic beads staining followed by magnetic separation. Lymph and Dermis DC were then cyto-centrifuged, fixed in PFA and reacted with anti-MHC-II (red) and anti-CD172a mAb (blue). Cells were further labelled for apoptotic DNA by the TUNEL method (green). Data are representative of 3 independent experiments.

In rat mesenteric lymph [25] and sheep oro-nasal and skin lymph [26], a CD172a^neg^ DC subset has been described that contains cytoplasmic apoptotic DNA. The pig skin derived CD172a^neg^ DC subtype (1) appeared CD11R3^low^CD163^neg^CD16^neg^CD207^neg^CD209^neg^ and strikingly CADM1^pos^ (Figure 2). It could be detected both in the dermis and the afferent lymph. We thus tested these DC for the presence of cytosolic apoptotic bodies. TUNEL^pos^ cytoplasmic inclusions were mostly found in the CD172a^neg^ DC from lymph and dermis (Figure 5). Moreover CD163 and TUNEL co-stainings showed the presence of apoptotic bodies uniquely into CD163^neg^ DC from lymph and dermis (data not shown). Finally no TUNEL staining was observed in LC (data not shown). These results indicate that transportation of apoptotic bodies from skin to lymph is a specific property of the CD172a^neg^CD163^neg^ dermal DC subpopulation in pig.

## Discussion

Using two markers selected for human and murine DC characterization, CD163 and CD172a, we described four DC subpopulations present in pig skin. Thanks to the new surgical protocol we developed, we were able to collect pseudo afferent lymph draining swine skin, and to observe that all the skin DC subsets we described did have the capacity to migrate in the lymph. Our goal was to define specific DC subtypes that could be targeted in skin vaccination. We thus considered of great importance to ascertain that each of these subtypes had the capacity to migrate through the lymph toward the LN, where they would eventually encounter the naïve lymphocytes in order to mount the desired immune response.

We more precisely characterized the already described [17] swine epidermal LC as CD163^neg^CD172a^pos^CD207^high^ which allowed us to identify their lymph-migrating counterpart, representing 11% of total afferent lymph DC. This subpopulation was also observed in the dermis, likely representing LC en route for the lymphatic. We defined three other DDC subpopulations: the CD163^neg^CD172a^neg^ DDC subtype (1) transports apoptotic bodies and is observed in equivalent proportion (∼20%) in swine and sheep skin lymph [26]. Its mouse (CD172a^neg^/CD103^pos^) [5], human (CD172a^neg^/BDCA3^pos^) [20] and sheep (CD172a^neg^/CD26^pos^) [21] counterparts are endowed with unique cross-priming capacities. Of note, the human BDCA3^pos^ DC have been observed in blood but have not yet been described in skin. Unfortunately neither CD103 nor BDCA3 nor CD26 antibodies are available in pig. CD163^neg^CD172a^neg^ DDC subtype (1) does not express CD207/Lang, at the difference with mouse CD172a^neg^/CD103^pos^ DC, whereas CD207 expression in BDCA3^pos^ human DC is still controversial [20], [27]. Conversely the sub population (3) defined as CD163^low^CD172a^pos^ presents a variable proportion of CD207^low^ cells. Except for CD207 and CD11R3, this sub population does not express other tested markers. Finally the CD163^high^CD172a^pos^ DDC population (2) expresses macrophagic markers such as CD209 and CD206 and might represents the equivalent of CD14^pos^CD163^pos^ human DDC [11], [12], [24].

Our unique pseudo-afferent catheterism technique allowed us to identify lymphatic counterparts to the dermal subpopulation we described. We can not rule out the possibility that these skin DC subpopulations modify their phenotype upon migration, however using a combination of several different markers present both on cutaneous and lymphatic DC, populations 1, 2 and 4 can be unambiguously distinguished in these two tissues. Population (1) is CD172a^neg^/CD163^neg^/CD16^neg^/CD206^neg^/CadM1^pos^, population (2) is CD163^high^/CD16^high^/CD206^high^ whereas, LC/population (4) is CD172a^pos^/CD163^neg^/CD16^pos^/CD207^high^/CadM1^pos^; in addition FITC painting revealed that at least one part of these lymphatic DC subpopulations originate from skin. Altogether these parameters plead for the fact that skin DC subpopulations 1, 2 and 4 might be the actual precursors of their lymphatic counterparts. We observed that the CD172a^neg^ population (1) is overrepresented in afferent lymph compare to dermis. Interestingly, a higher migration rate has been proposed to explain the higher percentage of their mouse (CD172a^neg^/CD103^pos^) counterpart in LN (13% of the DC) than in skin (5% of the DDC)[5]. Conversely, in swine, the CD163^high^ DDC population (2) is under represented in afferent lymph. These results might be interpreted by differences in their residence time in the tissue at steady state, the CD163^high^ DDC having a low turn over whereas the CD172a^neg^ DDC would have a more rapid turn over in the normal skin. It is important to note that we analyzed lymphatic DC present in the lymph more than 10 days post-surgery to eliminate the consequences of inflammation due to surgery. Conversely this work was performed on healthy conventional pigs, which might imply some background inflammation. Nevertheless, we consider these conditions comparable to what can be obtained when studying human skin samples [11], [12].

This work was aimed to define DC subpopulations in swine skin and to find their lymph migrating steady state counterparts. This first step accomplished, we are currently devising a transcriptomic study of the different swine skin subpopulations described here, in order to confirm their assignment to their murine, human [20] and sheep [21] counterparts. Moreover the definition of swine skin DC subsets and their assignment to their mouse counterparts will allow the design of subset targeting vaccines by the use of chimeric antibodies targeted to specific DC subsets. Antibody-based DC targeting has recently been shown to be exceptionally efficient in mouse models after a single vaccination (for review see [28]). For instance, in mice, targeting CD11b^high^ DC using DCIR2 [29], Dectin-1 [30] or CIRE/mDC-Sign [31] induced CD4 responses whereas targeting CD8α^pos^ spleen DC using anti-DEC-205 [29] or Clec9A [31] induced CD8 responses. Our description of swine DC subsets and the transcriptomic work in progress, will lead to the determination of target antigens specifically expressed on swine DC subtypes, allowing the development of this technology in swine which, in addition to its great interest in animal health, would be an important proof of principle for its translation in human medicine.

In conclusion, this study describes a skin DC network in swine similar to that of mouse and human. Finally the skin structure similarities between man and pig, now comforted by immunological homologies, allow us to propose the use of pig as a model of choice for the expanding field of transcutaneous vaccinations.

## Materials and Methods

### Ethics statement

This study was carried out under licenses from the Direction of the Veterinary Services of Versailles (accreditation n°A78-93, A78-15, A78-730). This study was approved by the Regional Paris South Ethics committee (n°08-001).

### Pseudo afferent lumbar lymph duct catheterism in miniature pigs

Pseudo-afferent lumbar lymph duct catheterization was performed in two steps. First, LN draining the flank, hindquarters, and inguinal areas were surgically resected. Two months later, after lymph vessel healing, a retro-peritoneal surgery was performed for inserting a silicone catheter (4 french, Nutricath bSQ, Vygon, Ecouen, France) in the lumbar trunk. The catheter was led out through a skin opening into a flask containing 500 units heparin. To facilitate lymph draining, pigs were left free in 2 m^2^ cages. Pigs received one intramuscular injection of terramycin (20 mg/kg, Pfizer, Paris, France) and of low molecular weight flunixin (Finadyne, 1 mg/kg, Schering Plough, Levallois Perret, France). Heparin (enoxaparin, Lovenox, Sanofi-Aventis, Paris, France) was injected intradermally every 12 h (1000 international unit anti-Xa) to prevent clotting. Mean outflow was of 9.1 ml/h, with 1.6.10^6^ cells/ml, and 0.7% of DC.

### Dendritic cells collection and purification

Lymph was collected twice a day. Cells were step-frozen in FCS containing 10% DMSO. Low-density lymph cells were obtained after centrifugation on a 1.065 density iodixanol gradient (Optiprep; Nycomed Pharma, Paris, France).

For FITC painting, FITC (Sigma, Saint-Quentin Fallavier France) was resuspended at 1 mg/ml in acetone/dibutylphtalate (V/V). The suspension was applied on the leg of the animal. Lymph cells were collected 2 days later and stained as described below.

Skin was obtained from swine undergoing surgical experiments. Skin biopsies were sampled using 8 mm punches (Stiefel, Wächtersbach, Germany). Skin biopsies were incubated overnight at 4°C in complete RPMI plus Dispase (Invitrogen, Cergy Pontoise, France). The next day biopsies, still in dispase, were incubated 2 h at 37°C allowing the separation of dermis and epidermis with tweezers. Epidermis and dermis were then incubated overnight at 37°C in complete RPMI (plus Collagenase D (Roche, Meylan, France) for dermis). Biopsies were filtered on 100 µm pores-nylon filters and cells were then processed for Facs staining.

For TUNEL staining, collagenase digested dermal or epidermal cells were first submitted to Ficoll, whereas lymph cells were deposed on optiprep gradiant. Enriched skin and lymph DC were then stained with anti-MHC-II (MSA3). Second step Miltenyi (Paris, France) anti-mouse antibodies coupled to magnetic beads were added, and cells were selected on column. MHC-II^pos^ cells represented routinely more than 80% of the purified cells.

### Antibodies and FACS Staining

The following antibodies were used: anti-CD1 (76-7-4), anti-CD172a (74-22-15, 74-22-15a), anti-MHC-II (Th21a, MSA3), anti-CD14 (CAM36) from VMRD (Pullmann, WA); anti-CD11R3 (2F4/11), anti-CD16 (G7), unlabeled or PE-coupled anti-CD163 (MCA2311) from AbDSerotec (Oxford, UK); anti-CD206 (122D2.08) and biotinylated-anti-CD207 (929F3.01) from Dendritics (Lyon, France); anti-CADM1 (3E1) from MBL (Woburn, MA), anti-CD21 (B-ly4) from BD/Pharmingen (Le Pont de Claix, France). The anti-CD209/DC-Sign is a kind gift from X.J. Meng [32].

Matched isotype controls for mouse IgG1, IgG2b, IgG2a were purchased from InVitrogen. Isotype controls for biotinylated rat-IgG2a and PE-coupled IgG1 were from BD/Pharmingen. Isotype-specific secondary reagents coupled to FITC, PE, Tricolor, alexa647, streptavidine-alexa488 were from InVitrogen. Dead cells were excluded by 7AAD (SIGMA) staining. Because 929F3 antibody recognizes an intracellular epitope, CD207 staining was done using CalTag Fix&Perm following manufacturer instruction. Cells were analyzed on a FACS-Calibur (BD/Pharmingen).

### Immunohistological staining of skin samples

Skin biopsies were snap-frozen in OCT (Sakura, Paris, France) and conserved at -80°C. Cryosections (5 µm) were cut using cryotom (Leica CM3050S, Nanterre, France). Sections were air-dried, fixed in cold methanol/acetone and stained using previously described anti-swine antibodies and isotype-specific secondary antibodies. Sections were mounted in SlowFade mounting medium (InVitrogen). Slides were examined on a LSM510 confocal microscope (Zeiss, LePecq, France), using a 40X, oil immersion objective.

### TUNEL staining of skin and lymph cDC

Magnetically enriched MHC-II^pos^ DC were deposed on slide by cytospin. Cells were air dried, fixed in 4%PFA before surface staining using previously described antibodies. Tunel staining was then processed according to manufacturer instruction (R&D, Lille, France).
